# Formative assessment of practical skills with peer-assessors: quality features of an OSCE in general medicine at the Heidelberg Medical Faculty

**DOI:** 10.3205/zma001335

**Published:** 2020-06-15

**Authors:** Andreas Möltner, Mirijam Lehmann, Cornelia Wachter, Sonia Kurczyk, Simon Schwill, Svetla Loukanova

**Affiliations:** 1 University Heidelberg, Baden-Württemberg Center of Excellence for Assessment in Medicine, Heidelberg, Germany; 2 University Heidelberg, Medical Faculty, Department of General Practice and Implementation Research, Heidelberg, Germany

**Keywords:** formative, OSCE, student examiners, generalizability theory

## Abstract

**Background: **Objective Structured Clinical Examinations (OSCEs) have become an established examination format at German medical faculties. Medical experts routinely use a summative assessment to evaluate practical and communicative skills, while the use of the OSCE format by student examiners, as a formative examination, remains rather limited.

**Objective:** The formative OSCE program of the Department of General Practice and Implementation Research at the Heidelberg Medical Faculty, which is conducted and evaluated by peer tutors, is examined with regard to its quality criteria and compared with summative OSCEs from other departments.

**Methods:** Difficulties and discriminatory power of individual testing stations were determined for the summative, as well as the formative OSCE, and compared with each other. To assess the reliability of the measurements, an analysis of the data was carried out using the Generalizability theory. In addition, a comparison is made between the assessments of student examiners and second assessments by medical experts.

**Results: **The stations of the formative OSCE show similar difficulties as those of the summative comparison OSCEs (P_form_=0.882; P_sum_=0.845 – 0.902). With respect to measurement reliability, there are no differences between the OSCE in General Medicine and the other subjects. The assessments of student examiners and medical experts correlate highly (r=0.888).

**Conclusion: **The formative OSCE in General Medicine is comparable to the summative comparison formats in terms of its quality criteria. The use of student examiners can be a reliable alternative to medical experts in formative OSCEs.

## Zusammenfassung

**Hintergrund: **Objective Structured Clinical Examinations (OSCEs) sind mittlerweile ein etabliertes Prüfungsformat an deutschen medizinischen Fakultäten. Üblicherweise werden darin praktische und kommunikative Fertigkeiten von medizinischen Experten summativ bewertet. Der Einsatz des OSCEs als formatives Prüfungsformat mit studentischen Prüfern findet bislang eher wenig Anwendung.

**Zielsetzung: **Der an der Medizinischen Fakultät Heidelberg im Fach Allgemeinmedizin durchgeführte formative OSCE, der von Peer-Tutoren durchgeführt und bewertet wird, soll hinsichtlich seiner Gütekriterien untersucht und mit denen summativer OSCEs aus anderen Fachbereichen verglichen werden.

**Methodik: **Schwierigkeiten und Trennschärfen der einzelnen Stationen werden für die summativen sowie den formativen OSCE bestimmt und einander gegenübergestellt. Zur Beurteilung der Messzuverlässigkeit wird eine Analyse der Daten mittels der Generalisierbarkeitstheorie durchgeführt. Zusätzlich findet ein Vergleich zwischen den Bewertungen der studentischen Prüfer und Zweitbewertungen medizinischer Experten statt.

**Ergebnisse: **Die Stationen des formativen OSCEs weisen ähnliche Schwierigkeiten wie die der summativen Vergleichs-OSCEs auf (P_form_=0.882; P_sum_=0.845 – 0.902). Bezüglich der Messzuverlässigkeit zeigen sich keine Unterschiede zwischen dem OSCE Allgemeinmedizin und denen der anderen Fächer. Die Bewertungen der studentischen Prüfer und der medizinischen Experten korrelieren hoch (r=0.888).

**Schlussfolgerung: **Der formative OSCE Allgemeinmedizin ist hinsichtlich seiner Qualitätskriterien vergleichbar mit denen der summativen Vergleichsformate. Der Einsatz studentischer Prüfer kann bei formativen OSCEs eine verlässliche Alternative zu medizinischen Experten darstellen.

## 1. Introduction

Practical clinical skills and anamnesis are already being taught at various medical faculties in the preclinical study semesters and tested with the help of an Objective Structured Clinical Examination (OSCE). It has been shown that an early learning of practical skills leads to better results in the clinical examination sections and clinical skills [1]. 

Traditionally, the teaching content is taught by faculty physicians, but increasingly also by student tutors of higher semesters. An advantage of peer tutors (Peer Assisted Learning, PAL) is the higher acceptance by students [2], lower costs [3], [4] and the possibility of smaller learning groups [5]. In addition, the students benefit from a reduction of stress and anxiety factors [6] and the student tutors [2], [7] benefit from their own in-depth study of the learning content. When comparing the student tutors with faculty members, the PAL students achieve the same results in final exams [8], [9], [10], [11] and the same or even higher quality of feedback [10]. Prerequisites for this are precisely defined student tutor training courses and checklists [12], [13].

Since 2013, practical skills and anamnesis techniques have been taught at the Heidelberg Medical Faculty in the pre-clinical part of the AaLPLUS courses (AaL: “Living Anatomy Plus”) of the Department of General Practice and Implementation Research with the help of peer tutors and subsequently examined in a formative OSCE, also conducted by student tutors [14]. A detailed description of the program and the evaluation of the OSCE by students and peer tutors can be found in [15].

Black and Wiliam [16] see five essential aspects of formative examinations. These are adapted to the context of University education:

1. Clarification and exchange of learning goals and success criteria
2. Initiating effective discussions and other learning tasks that demonstrate students' understanding of the learning content
3. Feedback that is useful for the students
4. Encouraging students to act as a mutual learning resource
5. Encouraging students to see themselves as initiators of their own learning activities

These objectives involve a whole process of teaching in which more or less continuously formative examinations are integrated. This is often logistically difficult to achieve fully in formative practical examinations in the form of OSCEs in medical education, so that the formative OSCE considered here should rather be seen as an instrument [17], which comes at the end of the pre-clinical part of the study. In order to achieve the goals announced by Black and Wiliam, other forms of formative examination procedures should be suitable [18]. Despite this limited function of the formative OSCE, it can be expected to have a positive effect on the learning behaviour of the examined students [19], [20].

In a review article by Khan et al. from 2017, 13 publications on the topic of “Students as examiners in OSCEs” are presented in more detail [21]. Some of the papers listed there examine the assessments of students and experts with regard to basic characteristics such as differences in the scores awarded and the correlation of the assessments of students and experts as examiners. A more detailed quantitative analysis, which also includes a differentiation of station- and examiner effects and their consequences for measurement reliability, is only provided in the works of Moineau et al. [10] and Basehore et al. [22]. In both studies, double evaluations at the stations by students and experts are investigated (in [22] the experts evaluated using videos of the examinations). However, it was not investigated whether student examiners differ from experts with regard to the extent of exam effects. 

Besides the comparison of student examiners and experts in the same formative examination, the quality of the formative examination in relation to the summative examinations established at the faculty is also of interest. Formative examinations differ in their objectives and structure (e.g. higher importance of feedback) and relevance of summative examinations to the examined students. The latter in particular, can have an effect on the reliability and accuracy of measurements, e.g. if the performance of the candidates is less differentiated due to reduced motivation.

### Aim of the study

The aim of the study was to demonstrate

1. that students in the context of formative examinations of practical skills can replace experts as examiners without compromising the quality of the examination and
2. that the quality of such formative examinations reaches the same standards as established summative examinations.

To this end, the formative OSCE in General Medicine at the Heidelberg Medical Faculty, which was held in 2018 and involved tutors as examiners, was examined with regard to its quality criteria (characteristics of the stations, measurement reliability of the exam, extent of examiner effects). A comparison was made with summative OSCEs, and a matching between the assessments of student examiners and those of experts (“supervisors”) was considered.

Other aspects of the formative OSCE in General Medicine with student examiners, such as acceptance by both examiners and examined students, assessment of the quality of feedback and subjective benefit to both students and examiners of the skills assessed in the OSCE are described in detail in [15]. The present study focuses exclusively on the quality characteristics of the OSCE that can be measured by statistical parameters of the examination results.

Standard analyses of tests usually include basic parameters such as difficulty, selectivity and reliability (see 3.1.1). Based on the Generalizability theory, the facets (influencing factors) “students” (differences in the ability of students), “station” (difference in the difficulty of stations), “examiner” (difference in the “strictness” of examiners) and the interaction “station x examiner” (different strictness of examiners at different stations) and their effects on generalizability and absolute measurement accuracy (see 3.1.2) were examined.

To compare the characteristic values of the OSCE General Medicine with established summative OSCEs of the Heidelberg Medical Faculty, the OSCEs of the subjects Surgery and Internal Medicine of the winter semester (winter term) 2017/2018, the summer semester (summer term) 2018 and the winter term 2018/2019 were used.

Finally, a comparison of double assessments by student examiners and experts within the formative OSCE General Medicine was conducted (3.2).

## 2. Methods

### 2.1. Implementation of the OSCE

The formative OSCE General Medicine in May 2018 was attended by 300 students of the fourth semester. The OSCE took place over two days and comprised four testing stations. One of the four stations (“venous blood sampling”) was completed by all students. Various clinical examinations had to be performed at two stations. These stations were not identical for the participating students, but alternated between the different parcours. A total of 11 different tasks were used (general examination of the abdomen, examination of spleen/kidney/appendicitis signs, blood pressure measurement, examination of the heart, liver, lymph node status, pulse status, thyroid gland, thorax, spine and a neurological examination). Furthermore, a complete anamnesis had to be taken. Here too, the contents changed (back, abdomen and head). Trained acting patients were used for the clinical examinations and the anamnesis. The contents of the stations and the essential criteria for evaluation were known to the participating students from the previous tutorials and given materials.

Each participant went through a total of four stations of eight minutes duration (5 minutes per task and 3 minutes feedback). The assessment of performance was carried out using checklists by students with basic didactic training who were at least in their sixth semester. A total of 25 points could be achieved at each of the stations. An exception to this were the three stations where an anamnesis had to be taken. In these, 30 points were to be achieved.

32 students were used as examiners, 26 of whom examined at several (up to five) stations during the course of the OSCE (see table 1 (Tab. 1)). The assessments were recorded using tablet computers (Programm tOSCE des UCAN-Prüfungsverbunds) [23].

Five supervisors were appointed to monitor the quality of implementation and evaluation, who carried out random second evaluation (135 evaluations in total). The trained examiners were (medical) staff members of the Department of General Practice and Implementation Research and, for the assessment of communicative skills at the three anamnesis stations, lecturers of the Department of Medical Psychology.

#### 2.2. Comparison with summative OSCEs

Six OSCEs of the subjects Surgery and Internal Medicine of the winter semesters 2017/2018 and 2018/2019 and of the summer semester 2018 of the Heidelberg Medical Faculty were used to compare the quality criteria of the OSCE General Medicine. The inclusion of several comparative OSCEs from two different subjects and semesters ensures that an estimate of the variability of their characteristic values (e.g. proportion of examiner influences) can be made for the comparative OSCEs. 

The OSCEs in Internal Medicine comprised 10 stations, those in surgery 13 stations. A maximum of 25 points could be achieved at all stations of these OSCEs (see table 2 (Tab. 2)). These OSCEs were performed on two to three days in two parallel courses (viz. “parcours”). The stations were partly changed in the different parcours. The two subjects Internal Medicine and Surgery were chosen because:

1. different examiners were used at the same testing stations and
2. the examiners were generally employed at different stations.

This allows an estimation of the examiner, stations and the interaction effect station x station during the evaluation.

#### 2.3. Statistical analysis

Difficulty P and corrected selectivities *r_it_* (correlations of the number of points achieved at one station with the points achieved at all other stations) as well as the mean inter-correlations with all other stations *r_ij_* (mean inter-item correlation) were determined for the stations of all mentioned OSCEs. The product-moment correlation (according to Pearson, two-tailed P value) was used throughout as a correlation measure.

In order to achieve equivalence of the stations, the point values obtained at the anamnesis stations, where 30 points were to be achieved, were rescaled to the range of 0-25 points for all analyses presented.

To assess the reliability of the measurements, the data were analysed using the Generalisability theory [24]. The facets considered were “students”, “stations”, “examiners” and the interaction “station x examiner”. From the variance components found by applying the Generalizability theory, the “generalizability” *Eρ^2^* (as an analogy to internal consistency/Cronbachs α) and the “dependability”* Φ* can be determined as a measure of absolute measurement accuracy:

If n denotes the number of stations, then


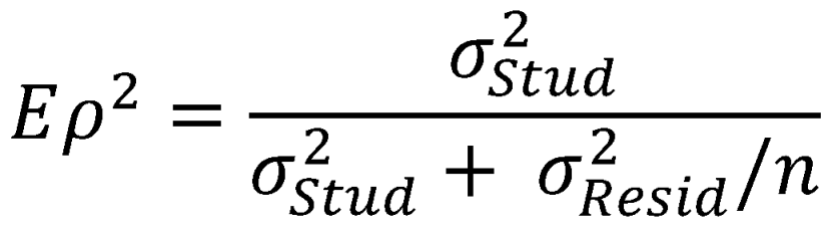
 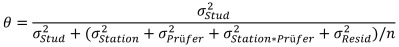


In order to analyse the matching between the assessments of the student examiners and the supervisors, the scores awarded for each station were compared (Wilcoxon signed-rank test) and the correlations determined. Furthermore, an analysis of variance of the total data set (examiners and supervisors) with the fixed factor “student examiner/supervisor” and the facets “students”, “stations”, “student examiners”, “supervisor” and the interaction “station x examiner” was carried out.

Note: When analysing with the Generalizability theory, a distinction must be made between so-called fixed and random factors. If the facet “student” is considered a random factor, the intention is to generalise to equivalent groups of students (i.e. in the same semester, same demographic composition, equivalent teaching etc.). The group of students considered in the examination being analysed should therefore be regarded as a sample from a population. The same applies to the facet “station”: As a random factor, the focus is on generalizability to equivalently constructed stations, while the facet “examiners” involves examiners from a potential group of examiners. When modelling the station or examiner as a fixed factor, however, the focus is on the stations or examiners actually used in the exam: Are individual stations particularly easy or difficult, are examiners too strict or too lenient? Since the present study focuses on generalizability, only the results for the analyses with “student”, “station” and “examiner” are presented as random factors.

The statistical analyses were performed with R Version 3.5.1. For the mixed model analyses for evaluation with the model of generalizability theory the packages “lme4” and “lmerTest” were used.

## 3. Results

### 3.1. Characteristic values of the test

#### 3.1.1. Difficulties and selectivity of testing stations

**Figure 1:**
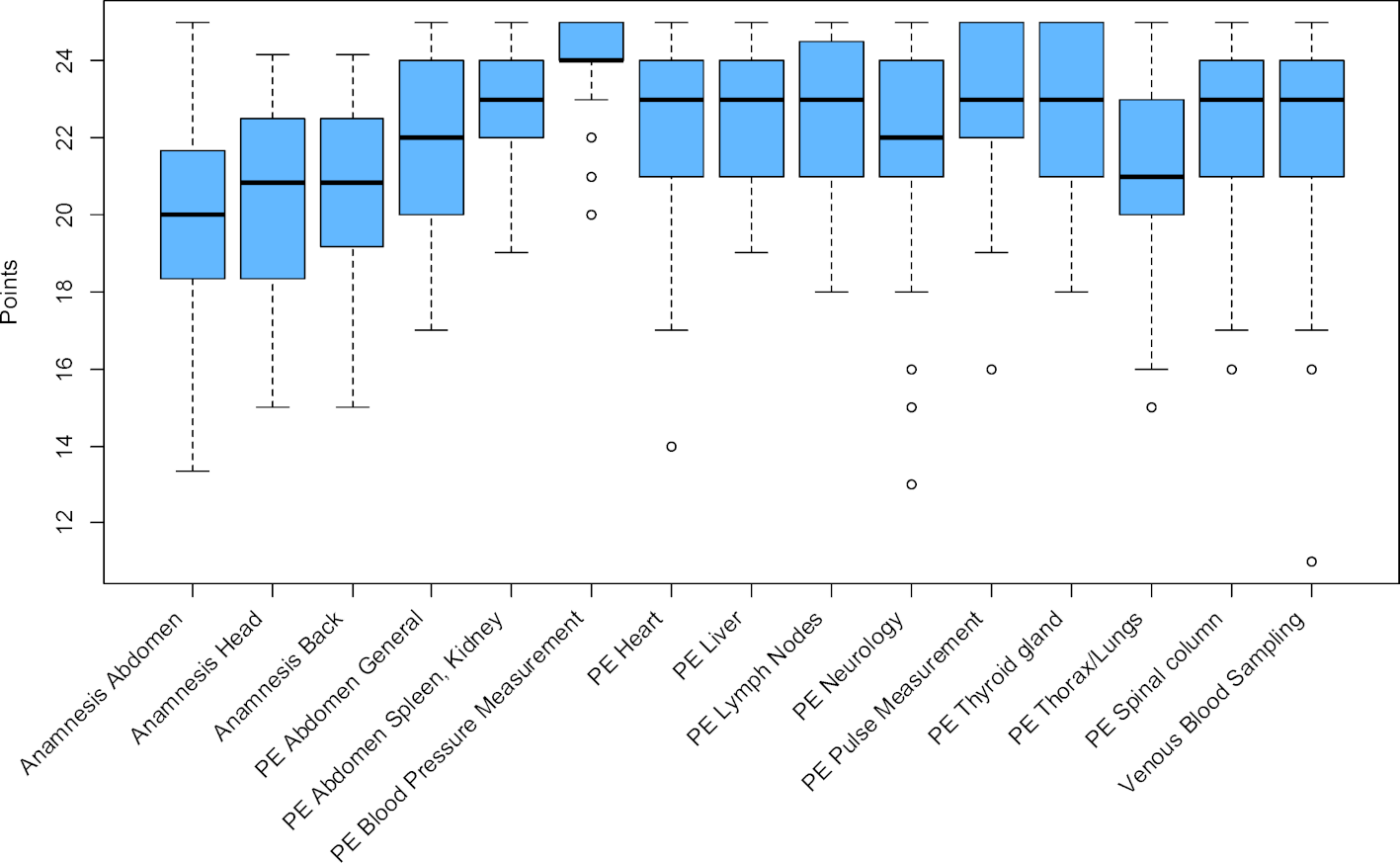
Distribution of the scores achieved at the stations of the formative OSCE General Practice. The station "Complete anamnesis", where 30 points were achieved in the original OSCE, has been rescaled to the range of 0-25 points.

The basic parameters (mean score achieved x, difficulty P and corrected selectivity rit) of the scores obtained at the stations are listed in table 3 (Tab. 3). Figure 1 (Fig. 1) contains a graphical representation of the distributions as a box plot.

The difficulties at the individual stations range from P=0.794 at the “Anamnesis Abdomen” station to P=0.959 at the “Blood Pressure Measurement” station. An average of 87.632 out of a maximum of 100 points was achieved. Please note that in contrast to dichotomous items, where only 0 or 1 point can be achieved, with finer granular evaluations (here 0-25 points) selectivities can possibly be interpreted even if the difficulties are numerically high. 

Eleven of the 15 stations have part-hole corrected selectivities of more than 0.300, two stations are just below this limit with selectivities of 0.276 and 0.296 (“Physical Examination Blood Pressure” and “Physical Examination Neurology”). Significantly lower are the stations “Physical Examination Liver” with *r_it_*=0.112 and “Pulse status” with* r_it_*=0.099.

##### Comparison with summative OSCEs

**Figure 2:**
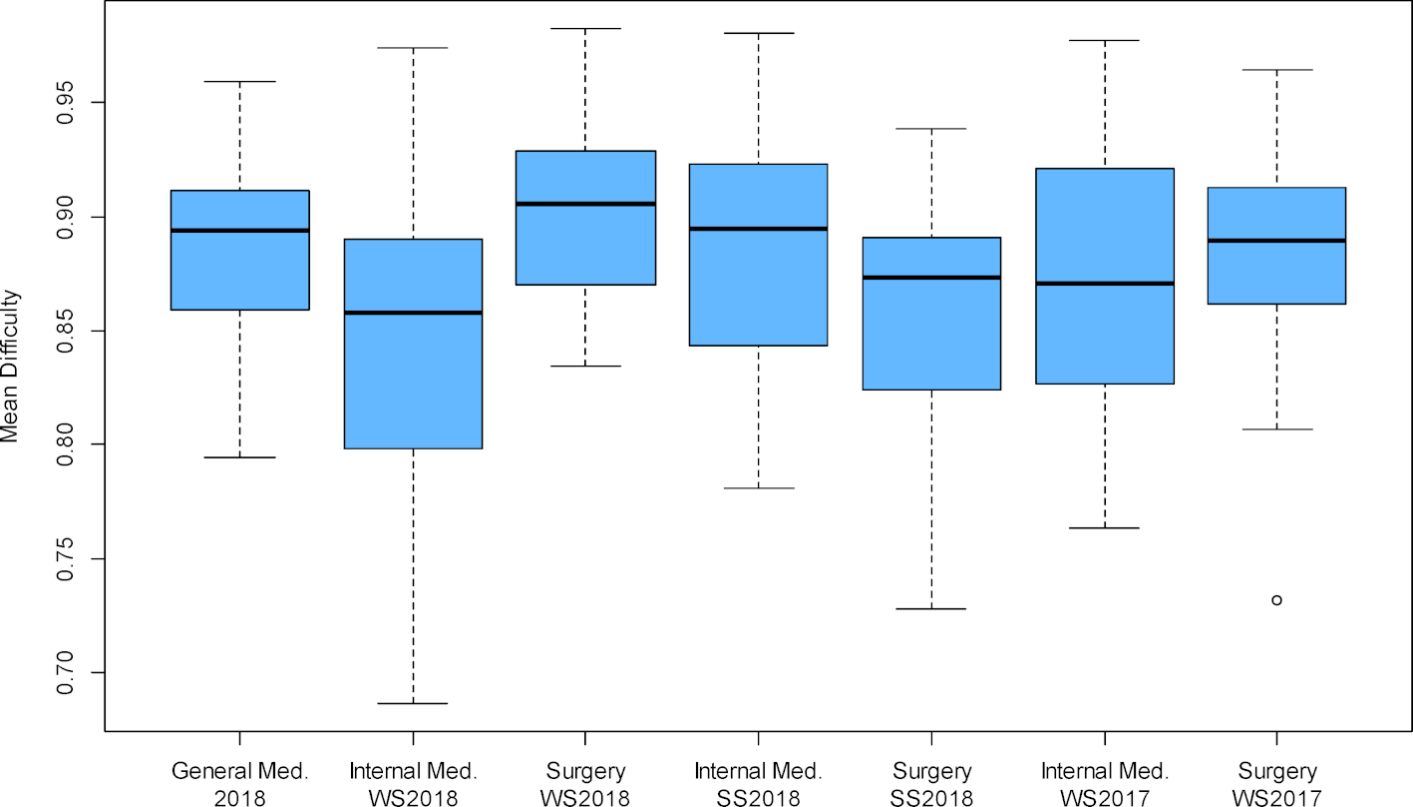
Distribution of the mean difficulties achieved P at the stations of the formative OSCE General Medicine 2018 and the summative OSCEs Internal Medicine and Surgery winter semester 2017/18 to winter semester 2018/2019.

Figure 2 (Fig. 2) shows the distribution of the scores achieved at the stations of OSCE General Medicine compared to the summative OSCEs in Internal Medicine and Surgery in the last three semesters (see also table 4 (Tab. 4)).

In comparison to the considered OSCEs of Internal Medicine and Surgery, the stations of the OSCE General Medicine were almost equally heavy (P=0.882 compared to P=0.876).

**Figure 3:**
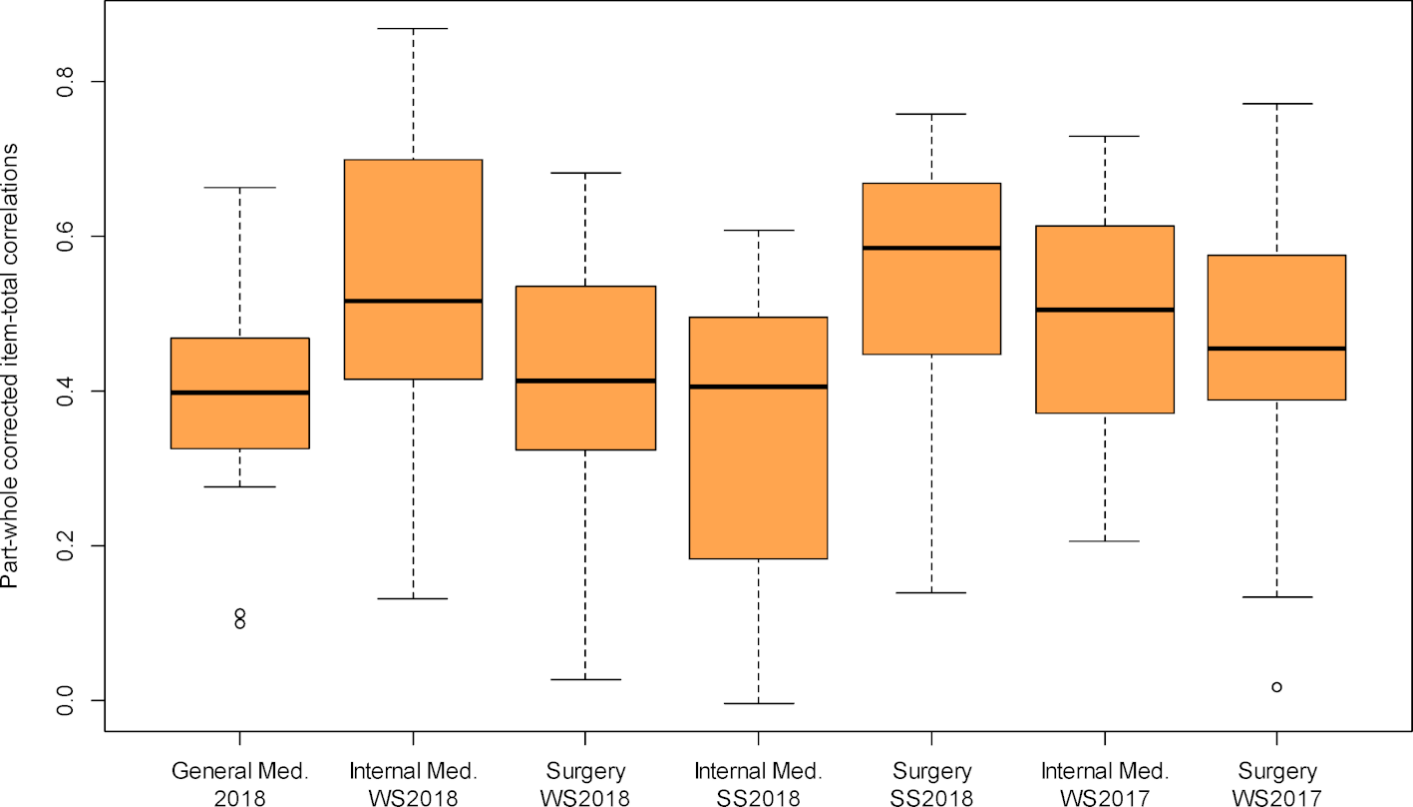
Distribution of corrected item-total correlations rit at the stations of the formative OSCE General Medicine 2018 and the summative OSCEs Internal Medicine and Surgery winter semester 2017/18 to winter semester 2018/2019.

**Figure 4:**
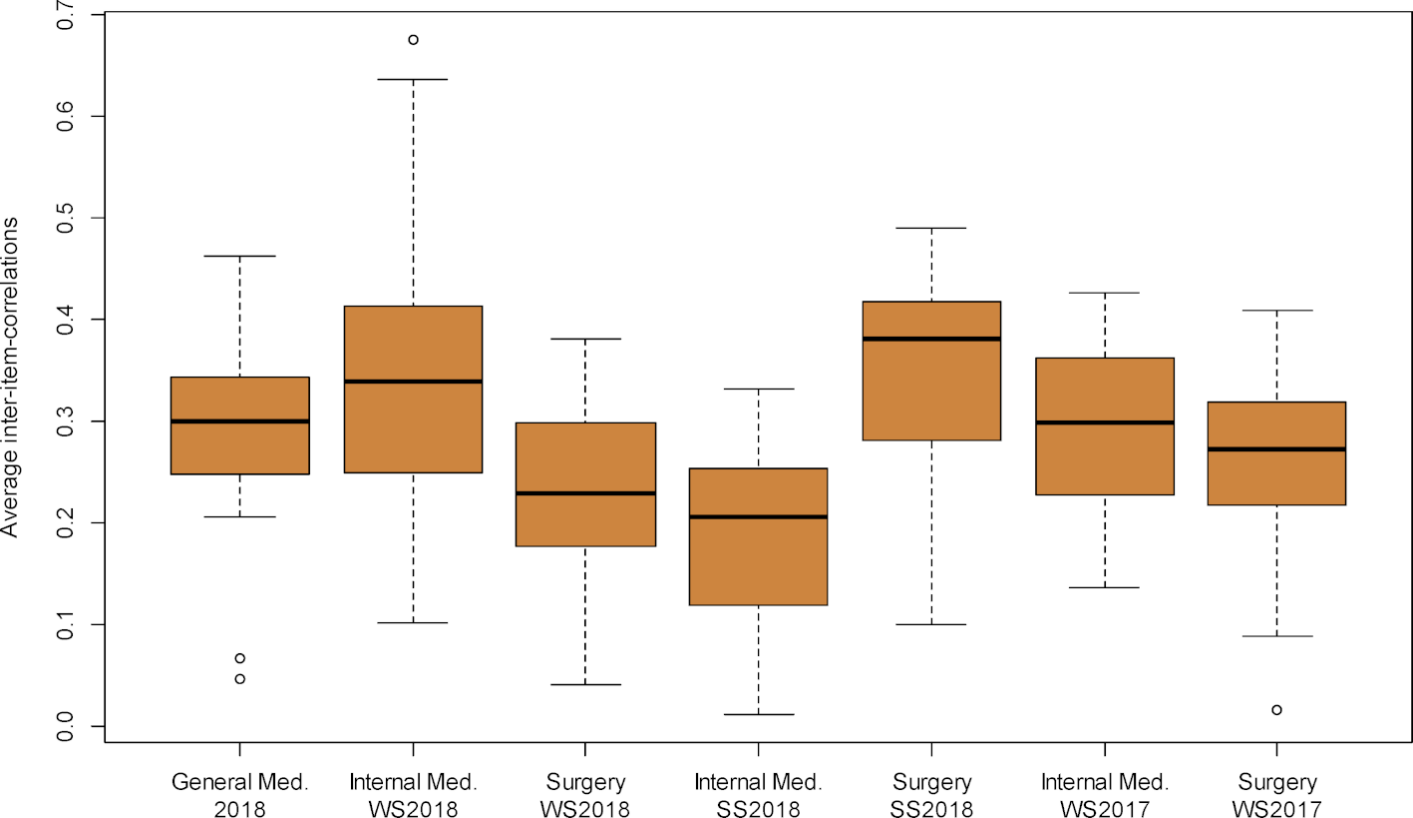
Distribution of averaged inter-item correlations *r_ij_* (correlations of the number of points achieved at one station with the respective other stations) of the formative OSCE General Medicine 2018 and the summative OSCEs Internal Medicine and Surgery winter semester 2017/18 to winter semester 2018/2019.

The corrected selectivities were on average lower than in the comparative OSCEs, only the OSCE Internal Medicine SS 2018 showed lower values (*r_it_*=0.358 compared to 0.386, see table 4 (Tab. 4) and figure 3 (Fig. 3)). In this comparison, however, it must be taken into account that in the OSCE General Medicine, the point total of the other stations used for the corrected selectivity is determined from only three stations, in contrast to Internal Medicine and Surgery with nine and twelve stations, respectively. This means that this sum is subject to more error variance in the OSCE General Medicine. A better possibility for comparison is offered here by the average of all correlations of the point sum from one ward with all other stations *r_ij_* (“mean inter-item correlation”). Here it can be seen that three of the comparison OSCEs each have lower and higher values (see table 4(Tab. 4) and figure 4 (Fig. 4)).

##### 3.1.2. Measurement reliability

Methods of Generalizability theory were used to analyse measurement reliability. A model with the facets “student”, “station”, “examiner” and the interaction “station x examiner” was analysed. Table 5 (Tab. 5) shows the estimated variance components of the facets.

Nearly 53% of the variance can be explained by the effects of the model, with 22% attributable to differences between students in terms of performance. The variability of the stations accounts for 21%, while the combined examiner influence was around 10%. The interaction effect station x examiner was not detectable or significantly different from 0.

The expected correlation of the point values achieved in the OSCE with an equivalent OSCE is *Eρ^2^*=0.647. These values do not take into account the effects of station and examiner, since in an equivalent parcours, all students pass through the same stations with the same examiners, so their total achieved points are only changed by these facets by a value that is constant for all and is not taken into account in a correlation (*Eρ^2^* is thus a measure of the relative measurement accuracy). In contrast, the Dependability Φ, as a measure of the absolute measurement accuracy, takes these factors into account, and is Φ=0.525 for the test.

##### Comparison with summative OSCEs

**Figure 5:**
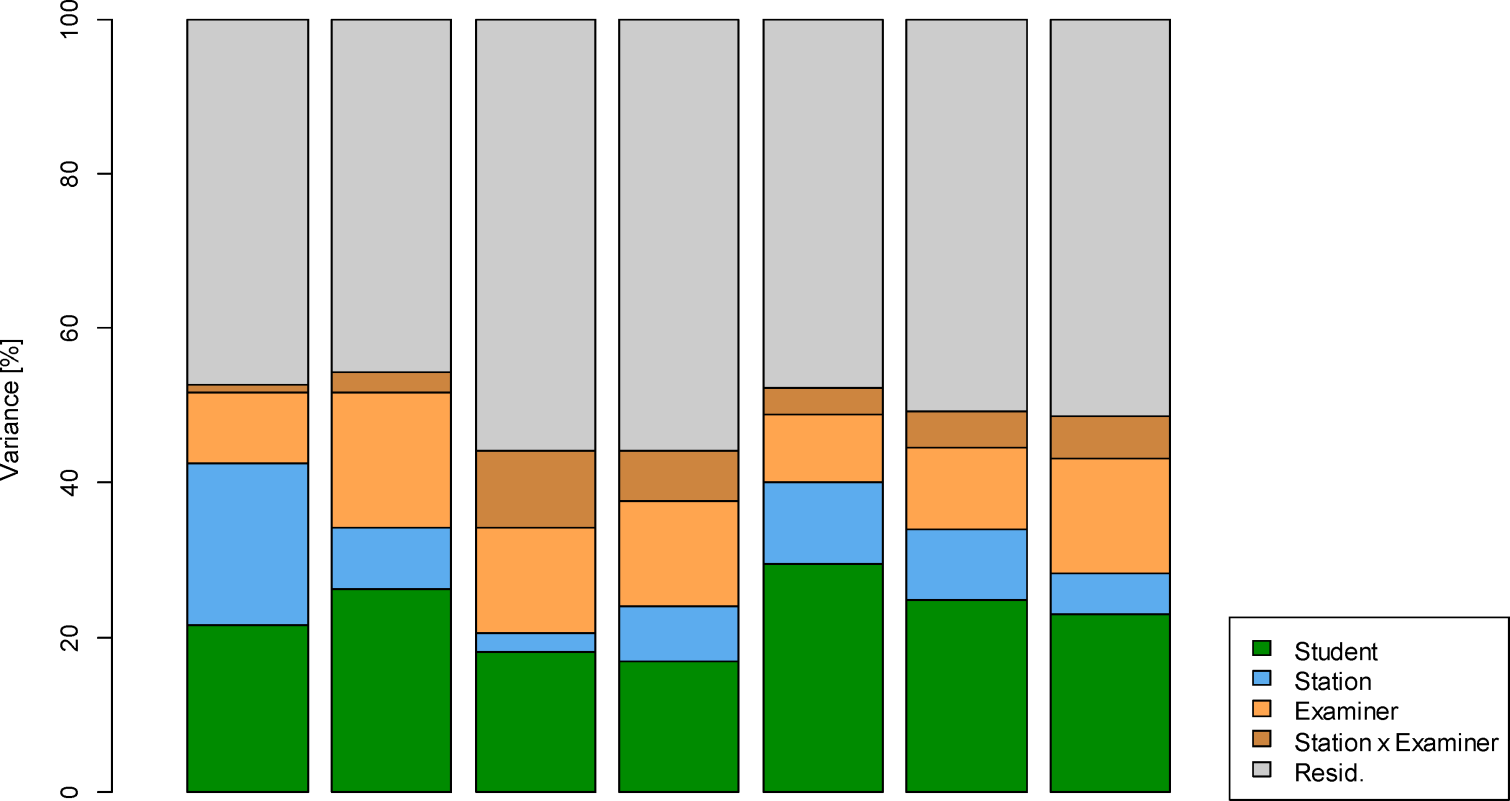
Percentage distribution of the variance of the OSCE General Medicine and the OSCEs Internal Medicine and Surgery from WS 2017 to WS 2018. The total variance is divided into the components "student", "station", "examiner", the interaction "station x examiner" and the residual variance.

Figure 5 (Fig. 5) shows graphically the percentage shares of the variance components for the OSCEs. A quality comparison of the OSCE General Medicine with those of Internal Medicine and Surgery with regard to the quality of the stations and the extent of the examiner’s influences must take into account the different number of stations. As an example, table 6 (Tab. 6) lists the values obtained on a Parcour with ten stations. It can be seen that for *Eρ^2^* three of the six comparison OSCEs have both lower and higher values. The absolute accuracy is higher for four comparison OSCEs. As can be seen in figure 5, this is mainly due to the higher variability of the stations.

#### 3.2. Supervision

In 135 assessments, an additional examination was carried out by a supervisor (medical staff of the Department of General Practice and Implementation Research and Medical Psychology), which serves as quality assurance of the OSCE (see table 1 (Tab. 1)). Table 7 (Tab. 7) shows the mean values of the assessments by the examiners, as well as those of the supervisors for the wards with double assessments. In addition, the significance value of the test for difference of assessments (Wilcoxon signed-rank test) is given. Only one station (“Anamnesis Abdomen”) shows a statistically significant difference.

**Figure 6:**
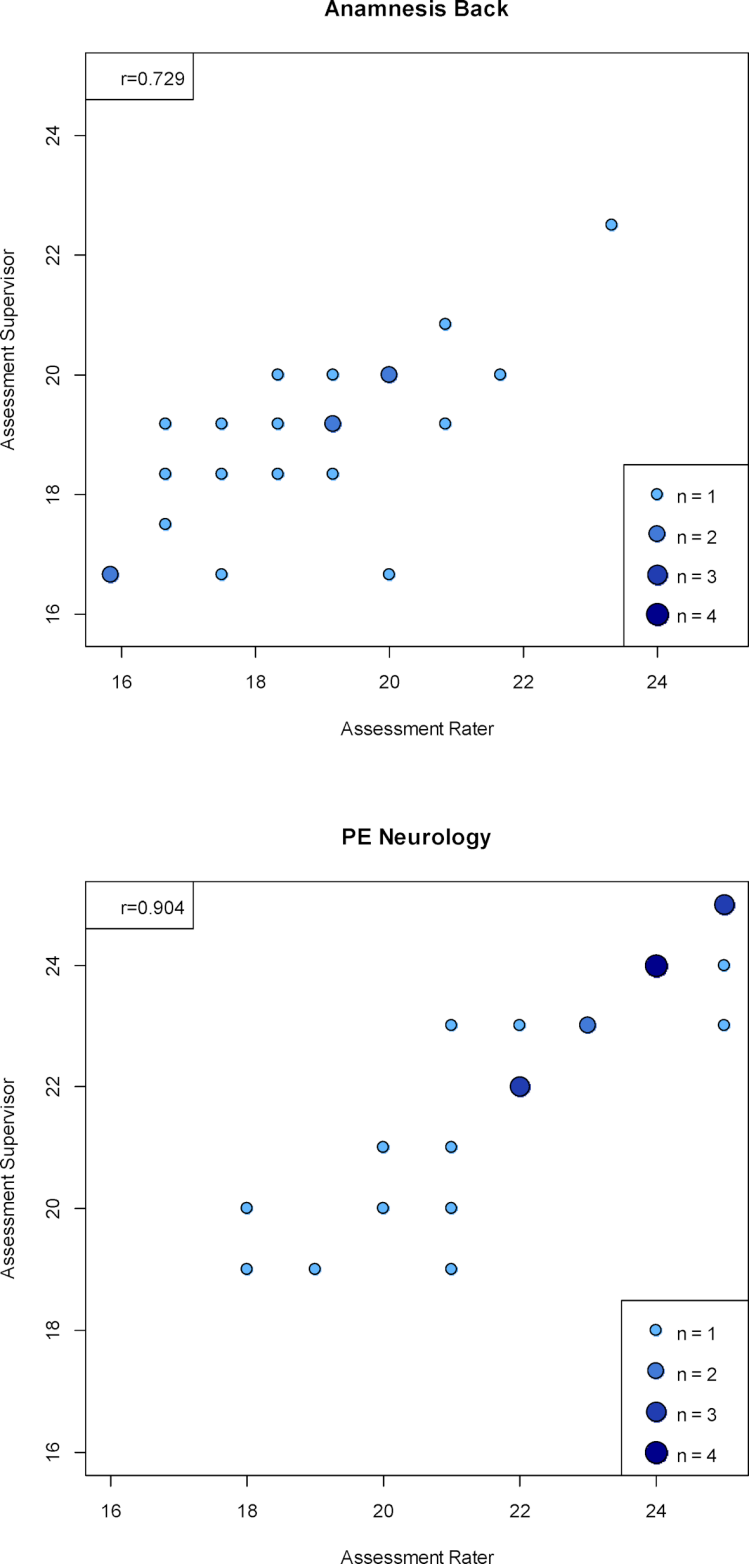
Scatter plots (bubble plots) of the assessments by examiners and supervisors at the "Back Anamnesis" and "Physical Examination Neurology" stations (the circle size represents the number of multiple data points with the same values).

Table 7 (Tab. 7) contains the correlations between examiners and supervisors at the stations, these ranged from 0.729 to 0.989. As examples, the scatter plots (bubble chart) of the assessments for the wards “Back Anamnesis” and “Physical Examination Neurology” are shown in figure 6 (Fig. 6).

An overall analysis based on the Generalizability theory of all data (student examiners and supervisors) with the examiner group as a fixed factor and with separate variance components for the two examiner groups is shown in table 8 (Tab. 8). The supervisors give 0.568 points less than the student examiners, but the effect is not significant (p=0.152). The examiner effects have a standard deviation of 0.700 points (see also table 5 (Tab. 5)). For the five supervisors, no variance component other than zero can be demonstrated (p=0.117), which is equivalent to the fact that no difference can be demonstrated with regard to their strictness.

## 4. Discussion

The results show that the stations of the OSCE General Medicine 2018 essentially fulfill the same quality criteria as the stations that are tested in the OSCEs in the subjects of Surgery and Internal Medicine, which have been established for years. In two of the physical examination stations, a review is recommended due to low selectivity. The matching of the assessments of the student examiners with those of the supervisors can be described as good to very good at all stations. Systematic differences between the assessments of the student examiners and the supervisors cannot be demonstrated. Although there is a relative influence of the examiners, the examiner effects tend to be even lower than in the comparison OSCEs. 

The generalizability standardized on ten stations is noticeably higher in the OSCE General Medicine with *Eρ^2^*=0.82 compared to the two studies mentioned in Khan's review [21], in which an analysis was carried out using the Generalizability theory, in [10] and marginally higher in [22] (*Eρ^2^*=0.51 for the checklist and *Eρ^2^*=0.63 for the “global score” and *Eρ^2^*=0.80 for the “total score”).

Apart from the number of stations, the measurement reliability of the OSCE examination in General Medicine is fully in line with the summative comparative OSCEs in the subjects of Surgery and Internal Medicine in the last three semesters.

This shows that with appropriate preparation:

1. students instead of experts can be used as examiners of practical skills and
2. the quality of a formative examination with student examiners is similar to that of established summative OSCEs with experts as examiners.

Since the implementation of practical format-based exams, which record the level of knowledge for students themselves as well as for teachers in a structured manner, often fails at the faculties due to the availability of examiners from the teaching staff, students in higher semesters offer a convenient alternative to substitute them.

The only weakness of the OSCE General Medicine is the small number of four stations that the examined students have to pass through. However, the fact that four stations does not provide a measurement reliability that meets the requirements of high-quality examinations is not surprising. This is in line with the literature, which demands significantly higher numbers of stations for OSCEs in order to obtain overall evaluations that can be classified as meaningful [25].

The analysis of other formative examinations in which students act as examiners is of course desirable, since it is not possible to generalize to other institutions, general conditions, or the like from the individual case presented here. Such investigations could show which conditions must be met for the use of student examiners in order to obtain statistically satisfactory and meaningful performance assessments. 

### Limitations

The random second assessment by the supervisors were not carried out systematically, so that the comparisons with the student assessors are partly based on very small data sets (see table 7 (Tab. 7)). There is also a room for improvement in the systematic allocation of the two physical examination stations from the set of eleven available stations among the examined students.

## 5. Conclusion

Overall, the OSCE General Medicine shows that it is possible to assess a large number of students with student examiners and thus to conduct high quality formative practical examinations. The involvement of students in the process of creating formative performance assessments is thus a practical way for medical faculties to take advantage of the widely recognized benefits of feedback in university teaching with the help of structured performance recording.

## Funding

The work was developed within the framework of the project MERLIN II (01PL17011C) funded by the Federal Ministry of Education and Research.

## Competing interests

The authors declare that they have no competing interests. 

## Figures and Tables

**Table 1:**
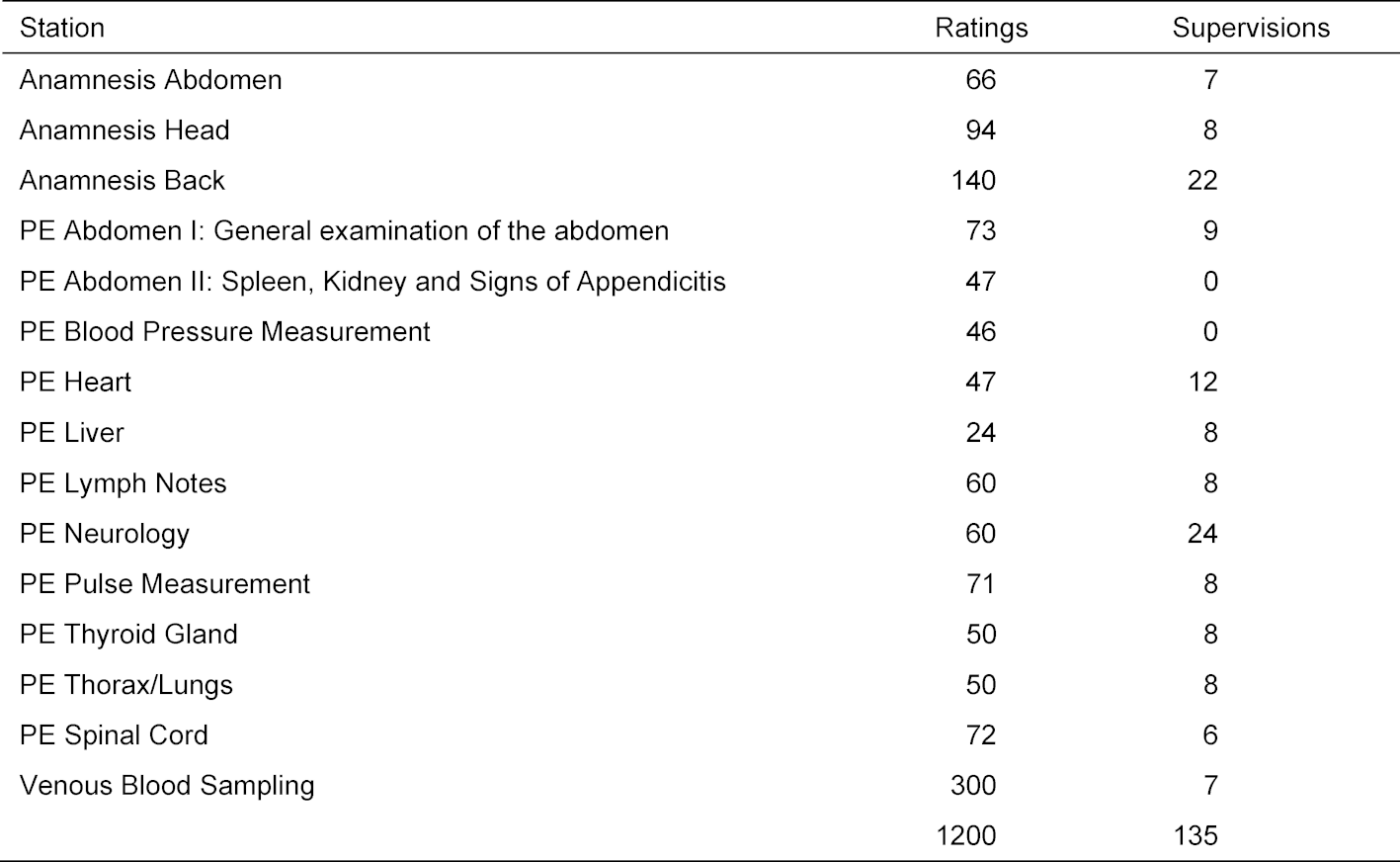
Number of assessments in the OSCE General Medicine 2018 by station (PE: Physical Examination).

**Table 2:**
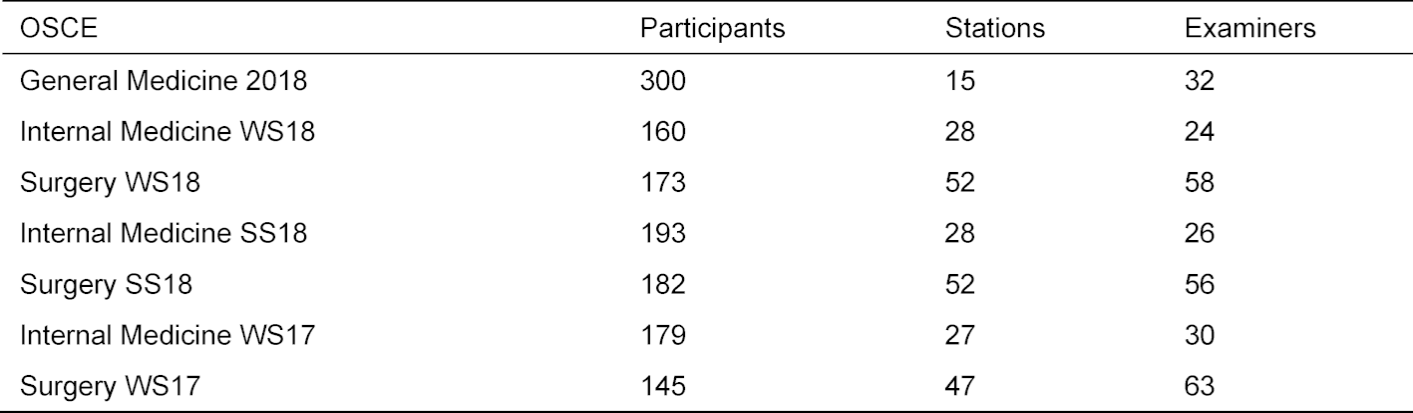
Number of participants, stations and examiners in the OSCE General Medicine and the OSCEs Surgery and Internal Medicine WS2017/2018 to 2018/2019.

**Table 3:**
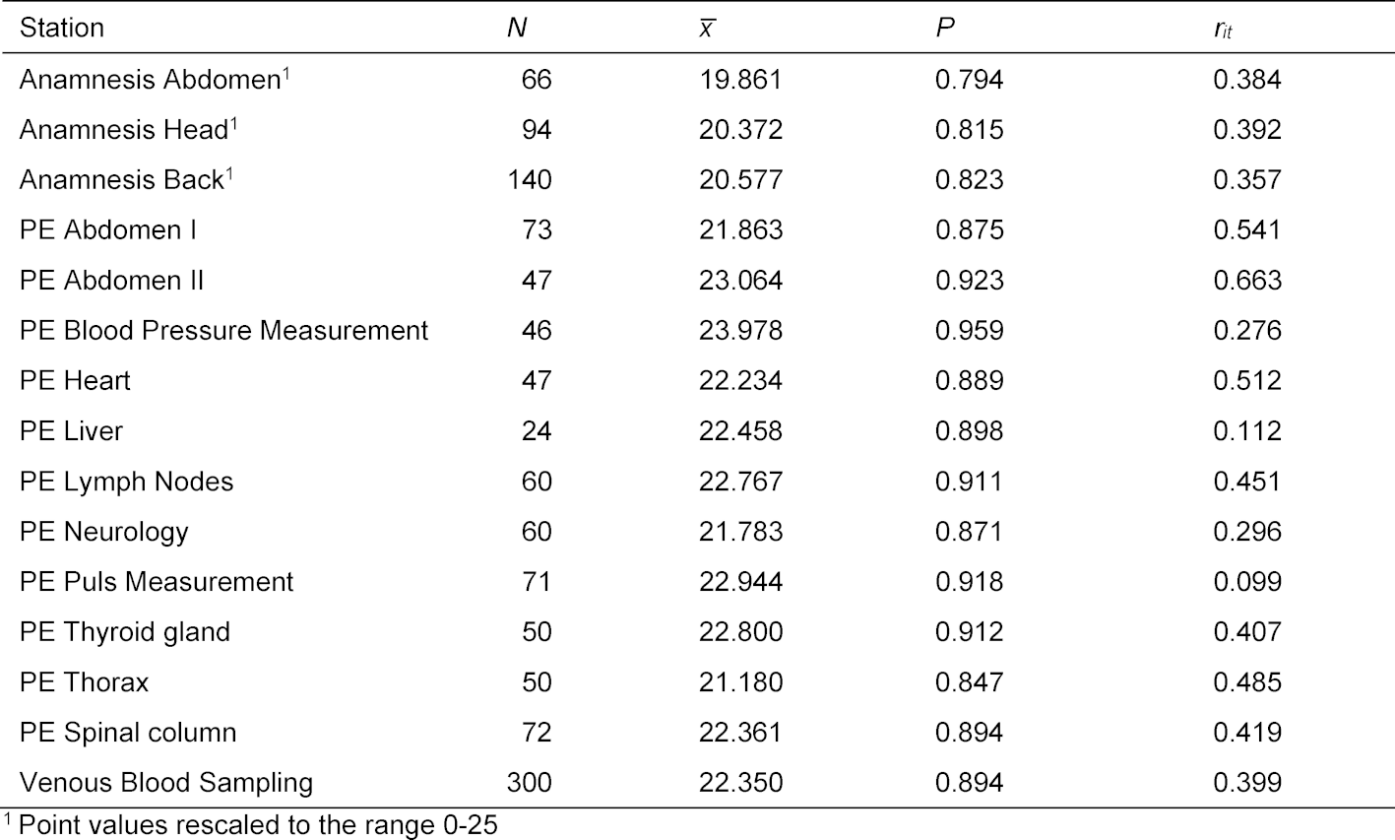
Characteristic values of the stations of the formative OSCE General Medicine 2018.

**Table 4:**
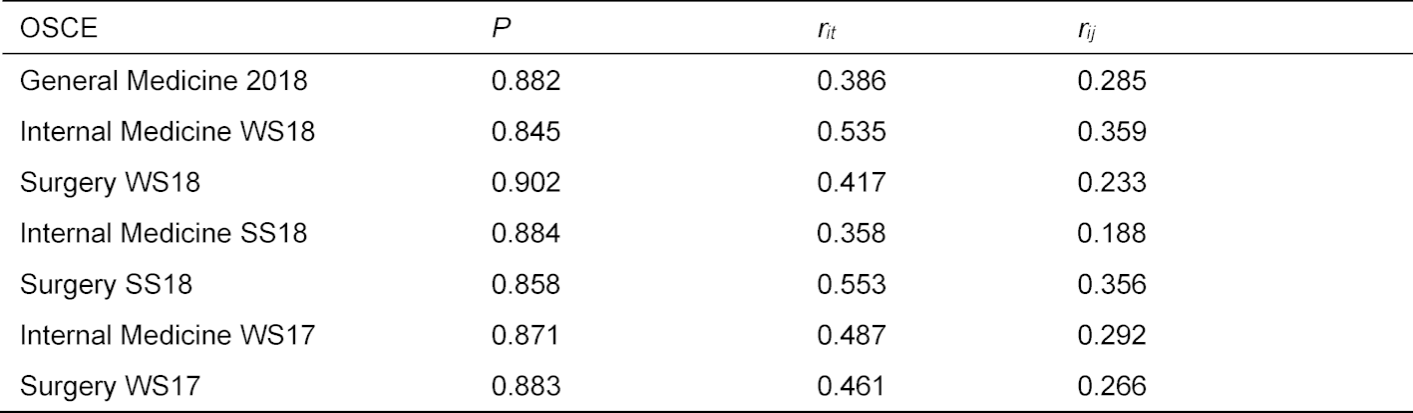
Average difficulties, discriminatory power and intercorrelations with other stations of the OSCE General Medicine and the OSCEs Internal Medicine and Surgery of WS 2017 – WS 2018.

**Table 5:**
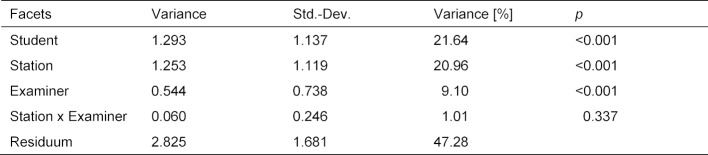
Variance components for the facets of the student, station, examiner and station x examiner. The standard deviation indicates the size of the influence of the respective effect in points at a station.

**Table 6:**
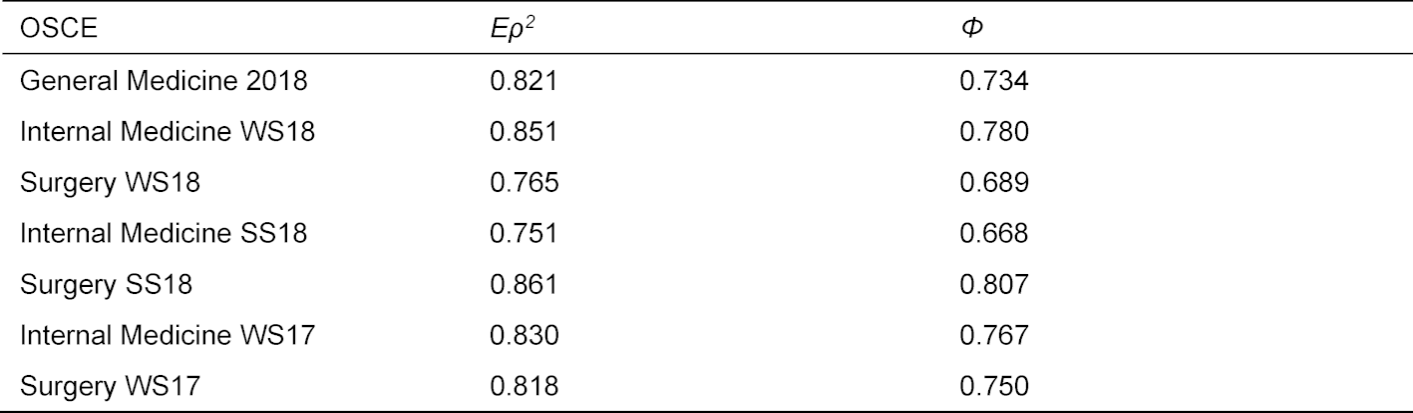
Estimated generalizability Eρ^2^ and dependability Φ for the OSCE in General Medicine and the OSCEs in Internal Medicine and Surgery, assuming a parcour of 10 stations.

**Table 7:**
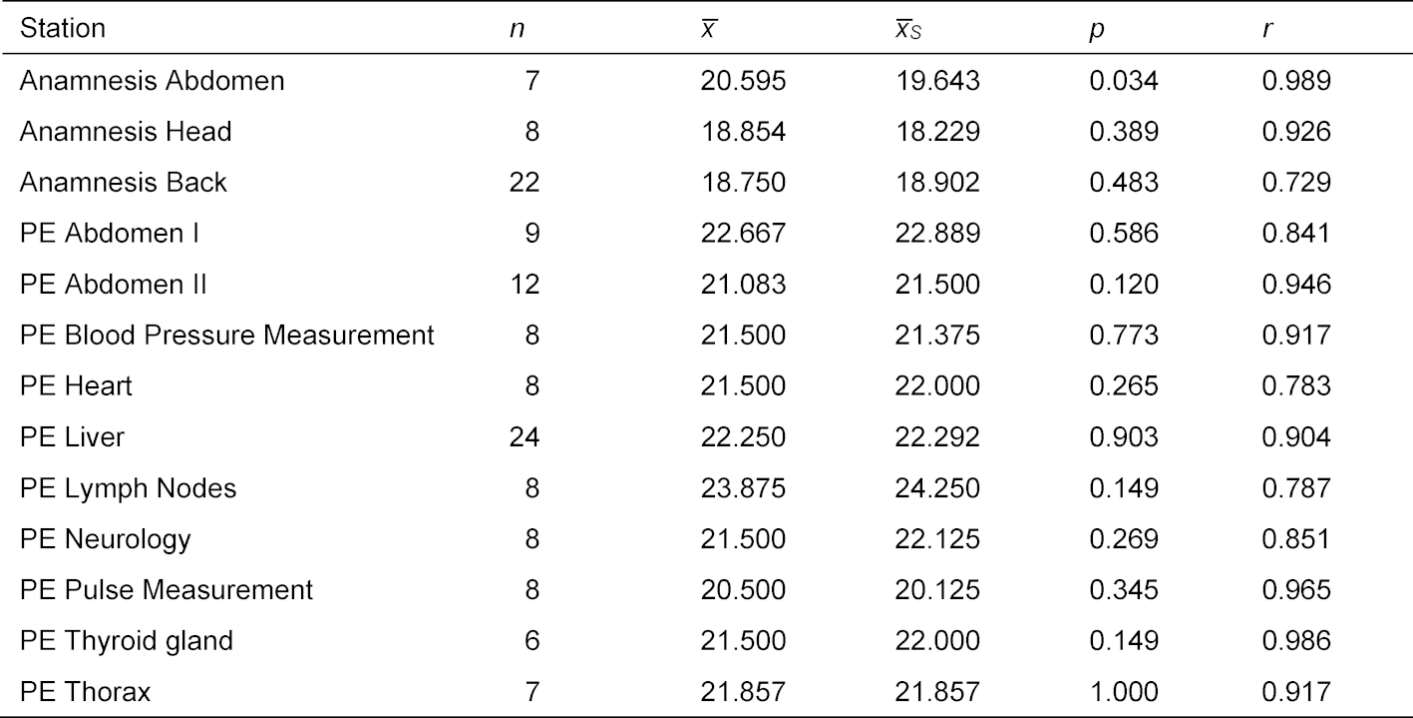
Comparison of assessments by examiners and supervisors: mean scores of examiners (x), mean scores of supervisors (xS), significance value of test for difference (Wilcoxon signed-rank test, p) and correlation of assessments (r).

**Table 8:**
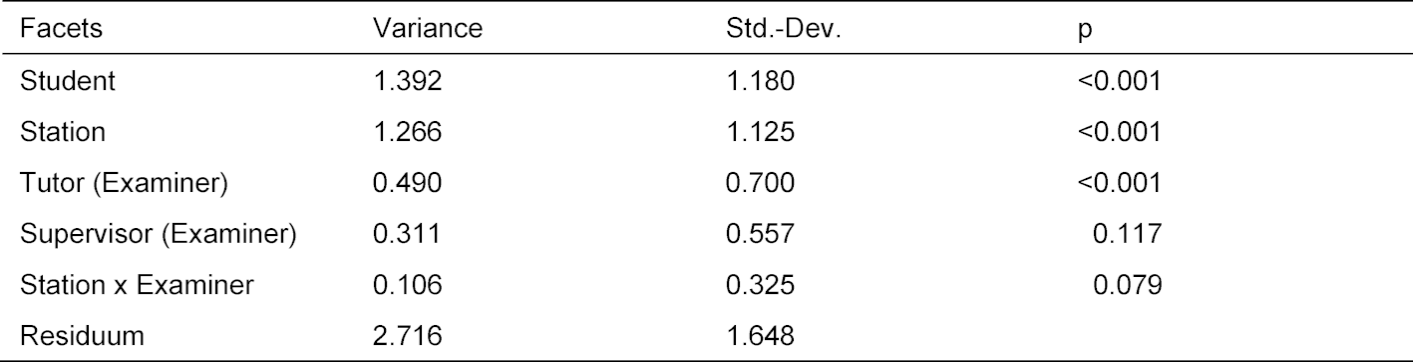
Variance components of the analysis of the assessments by student examiners and supervisors.
